# Case Series of Canine Myasthenia Gravis: A Classification Approach With Consideration of Seronegative Dogs

**DOI:** 10.1111/jvim.70113

**Published:** 2025-04-29

**Authors:** Rui Xavier Dos Santos, Jan Waelkens, Abbe H. Crawford, Sam Khan, Sara Sami, Sergio A. Gomes, Anouk Van Ham, Iris Van Soens, Ine Cornelis, Jake Canning, Joe Fenn, Patrick Waters, Sofie F. M. Bhatti, An E. Vanhaesebrouck

**Affiliations:** ^1^ Department of Veterinary Medicine University of Cambridge Cambridge UK; ^2^ Small Animal Department, Faculty of Veterinary Medicine Ghent University Ghent Belgium; ^3^ Department of Clinical Science and Services, Royal Veterinary College University of London London UK; ^4^ Nuffield Department of Clinical Neurosciences, Oxford Autoimmune Neurology Subgroup University of Oxford Oxford UK

**Keywords:** acetylcholine receptor, antibody test, autoimmune, junctionopathy

## Abstract

**Background:**

Myasthenia gravis (MG) is categorized into several subgroups, including seronegative MG. Seronegative human patients are well documented, but seronegative dogs remain clinically uncharacterized and their prevalence unknown.

**Objectives:**

This study aims to evaluate the clinical presentation, diagnosis, treatment, and outcome of canine MG subgroups.

**Animals:**

One hundred sixty‐seven owner‐owned dogs diagnosed with MG from three referral centers.

**Methods:**

Retrospective case series. We classified myasthenic dogs into subgroups, adhering to human guidelines.

**Results:**

We classified 167 dogs into four subgroups: acetylcholine receptor (AChR) antibody‐positive generalized (49.7%, *n* = 83/167), focal (19.2%, *n* = 32/167) and thymoma‐associated MG (9%, *n* = 15/167) and seronegative MG (22.2%, *n* = 37/167). Dogs with thymoma‐associated MG were older (median 102 months; Interquartile Range (IQR) 96–120; *p* < 0.001) and seronegative dogs were younger (median 30 months; IQR 11.5–66; *p* = 0.017), compared to the generalized subgroup (median 67 months; IQR 36–96). Seronegative dogs presented less frequently with megaesophagus, compared to the generalized subgroup (63.8% vs. 85.7%; Odds Ratio 3.4; 95% confidence intervals (C.I.) 1.4–8.9; *p* = 0.025). Myasthenic dogs' survival time was significantly reduced when thymoma (Hazard Ratio (H.R.) 3.7; 95% C.I. 1.4–9.9; *p* = 0.028) or esophageal weakness (H.R. 3.8; 95% C.I. 2.0–7.0; *p* < 0.001) was present. Conversely, a higher likelihood of remission was achieved when esophageal weakness was absent (H.R. 3.8; 95% C.I. 1.4–10.0; *p* = 0.007).

**Conclusion and Clinical Importance:**

Dogs with seronegative MG are more common than previously reported. Myasthenic subgroups differ in presentation and outcome, with esophageal weakness key to survival and remission. Diagnostic tests for seronegative dogs and effective treatments for esophageal weakness in myasthenic dogs are urgently needed.

**Abbreviations**
AChR: acetylcholine receptor
C.I.: confidence interval
O.R.: odd's ratio

## 1 Introduction

Myasthenia is a neuromuscular disorder characterized by fatigable muscle weakness. As defined in human medicine, it has two forms: congenital myasthenic syndromes, which are rare, caused by genetic defects in proteins involved in neuromuscular junction function [1] and acquired myasthenia or myasthenia gravis, which is more common, caused by autoantibodies against these proteins [2]. For more detailed information about canine myasthenia gravis, the authors refer to a recent veterinary review of Mignan et al. [3].

In humans, myasthenia gravis is categorized into subgroups based on the presence or absence of antibodies against the acetylcholine receptor (AChR) or other antigens, thymoma association, and age of onset [4]. These subgroups include AChR antibody‐positive generalized, ocular or thymoma‐associated myasthenia gravis, MuSK (muscle‐specific tyrosine kinase) and LRP4 (low density lipoprotein receptor‐related protein 4) antibody‐positive myasthenia gravis, and seronegative myasthenia gravis. The proteins MuSK and LRP4 are crucial for maintaining high AChR density at the neuromuscular junction [2]. Any disruption leads to myasthenia [5]. Each subgroup has implications for diagnosis, prognosis, and treatment [4, 6, 7].

Canine and human myasthenia gravis differ in several key aspects. Firstly, the focal, ocular form, which is common in humans, has not been reported in dogs. However, dogs do have a unique focal form that specifically affects their esophageal muscles. Unlike humans, dogs' esophageal muscles are striated or skeletal, not smooth [8]. Secondly, in veterinary medicine, we primarily test for antibodies against AChRs, with the RIA test being the most commonly used method. We do not routinely test for other potential autoantibodies like MuSK, although it has been briefly mentioned in the literature that MuSK antibodies have been found in one dog [9]. Currently, there is no commercial MuSK antibody test available for dogs.

In veterinary neurology, myasthenia gravis has historically been classified into three main forms: focal, generalized, and fulminant [10]. A recent veterinary review proposes categorizing myasthenia gravis into four subgroups: focal, generalized, fulminant, and seronegative myasthenia gravis, whilst distinguishing between dogs with and without thymoma [3]. While seronegative human patients are well‐documented, seronegative dogs remain clinically uncharacterized, and their prevalence remains unknown. The AChR antibody RIA test is currently the gold standard for diagnosing myasthenia gravis in dogs, with a reported 98% sensitivity [11]. This means that only 2% of myasthenic dogs test seronegative [11]. Its false positive rate is unknown. In contrast, AChR RIA detects antibodies in 80%–90% of human patients with generalized myasthenia gravis and in 50% of patients with focal myasthenia gravis [12, 13, 14, 15, 16], with a very low false positivity rate [17]. Based on our anecdotal clinical experience, we suspect the actual proportion of AChR RIA seronegative myasthenia gravis in dogs is higher than the reported 2% [11].

**Chart 1:**
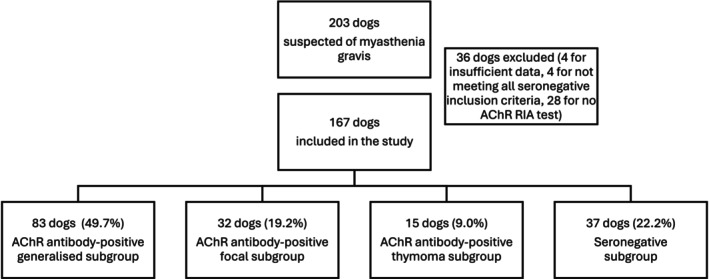
Classification of myasthenia gravis of 167 dogs of 3 centres.

For this study, we are implementing a classification system for canine myasthenia gravis based on the recent veterinary review of Mignan et al. [3], with adaptations to align it with the human classification system [4] (Chart 1). First, rather than treating the fulminant form as a distinct subgroup, we are using it as a severity grade. In dogs, fulminant myasthenia gravis corresponds to grade five or the most severe grade of muscle weakness on the human Myasthenia Gravis Foundation of America (MGFA) scale [18]. The MGFA scale is typically used to assess the severity of muscle weakness across all myasthenia gravis subgroups and a dog‐adapted scale will be employed in this study for this purpose. Secondly, we have categorized subgroups primarily by cause whether antibodies are present or absent rather than by clinical presentation (i.e., focal vs. generalized, followed by further division based on antibody status). Finally, we have consolidated the focal and generalized forms of myasthenia gravis associated with thymoma into a single category, termed “thymoma‐associated myasthenia gravis.”

This study aims to determine the percentage of dogs with seronegative myasthenia gravis and describe the clinical differences among the various myasthenia gravis subtypes.

## 2 Methods

### 2.1 Database Search

We conducted a search of veterinary databases from three referral centres. The search period varied from 1995 to 2024 for Ghent University, 2002 to 2023 for Cambridge University, and 2004 to 2024 for the Royal Veterinary College. We used clinical databases and the search terms ‘myasthenia,’ ‘mega(o)esophagus,’ ‘AChR RIA’ and generic and trade names of the various acetylcholinesterase inhibitor drugs, such as edrophonium (Tensilon), neostigmine (Prostigmin) and pyridostigmine (Mestinon) to identify dogs suspected of having myasthenia gravis. To be included in the study, dogs had to be diagnosed by a board‐certified neurologist or neurologist in training under the supervision of a board‐certified neurologist. All dogs were required to have had at least one AChR RIA test. Before data collection began, we designed a table to systematically record clinical information, including classification categories, using the definitions outlined below.

### 2.2 Classification System of Myasthenia Subgroups

For this study we used a classification system for dogs with myasthenia gravis that was based on a recent review [3], but has been further adapted to align with human classification guidelines. We classified myasthenia gravis into four subgroups: AChR antibody‐positive generalized (further referred to as generalized subgroup), AChR antibody‐positive focal (further referred to as focal subgroup) and AChR antibody‐positive thymoma‐associated myasthenia gravis (further referred to as thymoma subgroup), and seronegative myasthenia gravis (further referred to as seronegative subgroup; Chart 1). For inclusion in the generalized subgroup, dogs had to exhibit both generalized fatigable muscle weakness and a positive AChR antibody RIA titre (> 0.6 nmol/L). For the focal subgroup, dogs had to show megaesophagus confirmed by thoracic radiographs, or esophageal hypomotility confirmed by videofluoroscopic swallowing study and a positive AChR antibody RIA titre [19]. While dogs within the focal subgroup may also present with additional facial, laryngeal, or pharyngeal signs, they have no generalized signs of fatigable muscle weakness, such as limb weakness. If a dog initially exhibited focal signs and subsequently developed generalized signs, the dog was included in the generalized subgroup. For the thymoma‐associated subgroup, dogs had to exhibit signs of either generalized or focal fatigable muscle weakness, along with evidence of thymoma on thoracic radiography or CT with cytology or histopathology, and a positive AChR antibody RIA titre. For the seronegative subgroup, by analogy with the human definition [15, 20], dogs had to exhibit fatigable muscle weakness and a decrement of more than 10% on repetitive nerve stimulation or a clear improvement in fatigable muscle weakness after acetylcholinesterase inhibitors (such as edrophonium, neostigmine or pyridostigmine), combined with a negative AChR RIA titre (< 0.6 nmol/L). Improvement was determined by a noticeable increase in walking distance without rest, resolution of megaesophagus on radiographs, or resolution of decrement on repetitive nerve stimulation. If the clinician recorded the response to acetylcholinesterase inhibitors as ‘unclear,’ it was considered negative. Dogs exhibiting signs of myasthenia that manifested before the age of 4 months or with a family history of other littermates affected but without a positive AChR titre were excluded, as they were more likely to have congenital myasthenic syndrome [3].

### 2.3 Myasthenic Signs

The clinical records were reviewed to identify the presence of the following clinical signs, such as fatigable muscle weakness and muscle atrophy. Fatigable muscle weakness was further categorized into general, facial, laryngeal, pharyngeal, and esophageal weakness. Generalized fatigable muscle weakness due to myasthenia can manifest in various ways. In most cases, the animal would be able to walk for a few meters after resting and take a few steps before sitting or lying down. However, in severe cases, muscle weakness could progress to non‐ambulation, accompanied by severe respiratory weakness (myasthenic crisis). Facial weakness was characterized by fatigable muscle weakness observed during the palpebral reflex. Laryngeal weakness was defined as a change in bark or laryngeal stridor. Pharyngeal weakness was defined as dysphagia. Esophageal weakness was defined as regurgitation. Muscle atrophy, which included both masticatory and generalized muscle atrophy, was based on subjective observation.

### 2.4 Severity Scoring of Myasthenic Signs

Based on clinical records, we retrospectively assigned a severity score to generalized, facial, and bulbar muscle weakness using a modified MGFA classification system [18]. Bulbar muscle weakness is a human term that describes muscle weakness of the larynx, pharynx, and esophagus.

Generalized and facial weakness was scored as follows:

0. No generalized or facial weakness,
1. Pure facial muscle weakness,
2. Mild generalized weakness (increased effort, but no rest required for independent ambulation),
3. Moderate generalized weakness (rest required for independent ambulation),
4. Severe generalized weakness (assistance required for ambulation),
5. Myasthenic crisis (which causes or threatens respiratory failure)/intubation/death due to myasthenia gravis‐related complications. Grade 5 corresponds to ‘fulminant myasthenia gravis’.

Bulbar weakness was scored as follows:

1. No bulbar signs,
2. Mild bulbar signs (rare cough during swallowing or rare regurgitation),
3. Moderate bulbar signs (frequent choking or regurgitation),
4. Severe bulbar signs (requiring feeding tube),
5. Death due to myasthenia gravis‐related complications.

### 2.5 Diagnostic Testing

AChR antibody testing was performed using RIA, as previously established [21]. At least one RIA test was available for each dog. A decrement was defined as a reduction in Compound Muscle Action Potential of more than 10% during a series of 10 supramaximal stimuli of 1–5 Hz [22]. All thoracic radiographic images were reviewed by board‐certified radiologists. Megaesophagus was diagnosed based on diffuse dilatation of the esophagus on conscious radiographs, while aspiration pneumonia was diagnosed by observing a cranioventral interstitial or alveolar lung pattern on thoracic radiographs. Esophageal hypomotility was confirmed in some dogs by video fluoroscopic swallowing study (for criteria, see [23]).

### 2.6 Outcome

For some dogs, outcome data were available from recorded telephone or email communications with owners or from the clinical database, including cases where dogs died in the hospital. If needed, we contacted referring veterinarians to obtain clinical records with the owner's permission. When clinical records were unavailable, we reached out to owners by phone.

The time of survival was calculated as the duration between the time of diagnosis and the animal's death. For calculating Kaplan–Meier or survival curves, we defined an ‘event’ as death related to myasthenia gravis, including death to side effects of medications or complications. Animals were ‘censored’ if they were still alive or died due to a non‐myasthenia gravis‐related cause. Cases with no outcome data (e.g., very old cases) were excluded from survival analysis.

To calculate the remission rate, we used the term ‘complete stable remission,’ which is defined as the disappearance of all myasthenia gravis signs without any treatment [24], accompanied by the persistence of symptom‐free status for at least 3 months. The time of remission was calculated as the duration between the time of diagnosis and remission. To calculate Kaplan–Meier curves for remission in myasthenia gravis, we defined an ‘event’ as remission. Animals were ‘censored’ at their last follow‐up time when they were not in remission.

Statistical analysis compared the four subgroups for differences in clinical signs, severity of clinical signs, and AChR antibody levels. For continuous variables, ANOVA was used when the residuals were normally distributed (with Welsh correction if unequal variances), and the Kruskal‐Wallis test was used otherwise. For categorical variables, cross tables and Chi‐square tests were employed. To compare the severity of myasthenia over time across the four subgroups, a linear mixed model was employed: the animal was treated as the intercept, the severity score was considered the dependent factor, and time was treated as a fixed factor. The residuals obtained from the linear mixed model were checked for a normal distribution. We compared each subgroup against the generalized subgroup and applied Dunnett's correction to account for multiple comparisons. To test for survival and remission, a log‐rank test was used.

Continuous data is presented in Tukey box plots, categorical data is displayed in a table, and survival data is illustrated using a Kaplan–Meier survival plot. SPSS and Prism software was employed for both data analysis and graphing. Data was considered statistically significant if the *p*‐value was less than 0.05. Significance in graphs is indicated by significance bars. All tests were conducted with a two‐tailed approach.

## 3 Results

### 3.1 Dogs Studied

Two hundred three dogs from three centres were suspected of having myasthenia gravis. Thirty‐six dogs were excluded from this study (Chart 1). Four dogs were excluded due to incomplete clinical records (2 from centre 1, 1 from center 2, 1 from centre 3). Four dogs (all from center 3) were excluded from the seronegative group because they did not meet the inclusion criteria. Specifically, they did not receive pyridostigmine (3 dogs) or did not show a beneficial response to it (1 dog) and repetitive nerve stimulation was not performed. The remaining 28 dogs were excluded because no AChR test was performed (3 from centre 1, 16 from centre 2 and 9 from centre 3). This was usually because these dogs had died or been euthanized shortly after presentation, so the AChR test was not performed or canceled for these dogs. This resulted in a final cohort of 167 dogs that met the inclusion criteria. Forty dogs came from centre 1, 61 dogs came from centre 2, and 66 dogs came from centre 3.

### 3.2 Proportion of Subgroups

Eighty‐three dogs (49.7%) met the criteria for AChR antibody‐positive generalized myasthenia gravis, while 19.2% had AChR antibody‐positive focal myasthenia, and 9.0% had AChR antibody‐positive thymoma (Chart 1). Twenty‐two percent of the dogs were seronegative. The proportion of seronegative individuals varied across the three centres: centre 1 had 27.5% (*n* = 11/40), centre 2 had 23.0% (*n* = 14/61), and centre 3 had 18.2% (*n* = 12/66).

### 3.3 Signalment

**FIGURE 1:**
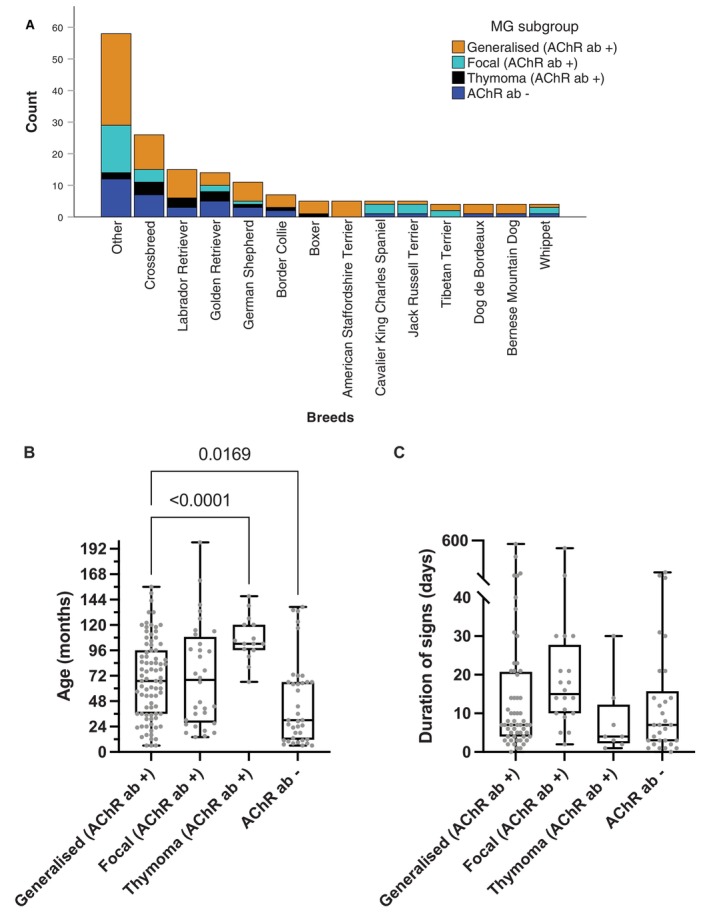
Signalment. (A) Breed distribution. Breeds less than 2% were categorized within ‘other breeds’. (B) Age of onset of clinical signs was analyzed for the 4 myasthenic subgroups. (C) Duration of clinical signs prior to presentation was analyzed for the 4 myasthenic subgroups.

Fifty‐four distinct breeds were included in the study (Figure 1A). The overall male‐to‐female ratio was 1.29:1 (Table 1).

**TABLE 1:** Clinical presentation of the 4 different myasthenia gravis subgroups in 167 dogs.

Proportion of dogs	Subtype				*p*	Total
	AChR ab + generalized	AChR ab + focal	AChR ab + thymoma	AChR ab−		
Sex (M/F)	1.24/1 (46/37)	1.67/1 (20/12)	1.14/1 (8/7)	1.18/1 (20/17)	ns	1.29/1 (94/73)
Neutering (neutered/intact)	42/41	14/18	4/11	16/21	ns	76/91
Generalized weakness (%, *n*)	100.0 (83/83)	0.0 (0/32)	93.3 (14/15)	91.9 (34/37)	N/A	78.4 (131/167)
Facial weakness (%, *n*)	47.0 (39/83)	40.6 (13/32)	40.0 (6/15)	32.4 (12/37)	ns	41.9 (70/167)
Laryngeal weakness (%, *n*)	24.1 (20/83)	12.5 (4/32)	20.0 (3/15)	27.0 (10/37)	ns	22.2 (37/167)
Pharyngeal weakness (%, *n*)	53.0^‡^ (44/83)	37.5 (12/32)	46.7 (7/15)	21.6^‡^ (8/37)	^‡^0.004	42.5 (71/167)
Esophageal weakness (%, *n*)	84.3 (70/83)	100 (32/32)	100.0 (15/15)	67.5 (25/37)	ns	82.1 (96/167)
Muscle atrophy (%, *n*)	1.2^#^ (1/83)	3.1 (1/32)	13.3^#^ (2/15)	5.4 (2/37)	^#^0.036	3.5 (6/167)
Megaesophagus (%, *n*)	85.7* (66/77)	100.0 (31/31)	92.3 (12/13)	63.8* (23/36)	*0.025	84.1 (132/157)
Aspiration pneumonia (%, *n*)	53.2 (41/77)	64.5 (20/31)	69.2 (9/13)	47.2 (17/36)	ns	55.4 (87/157)
Decrement (%, *n*)	47.8 (11/23)	37.5 (3/8)	66.7 (2/3)	46.2 (6/13)	ns	46.8 (22/47)
Beneficial response to AChEI (%, *n*)	93.4 (57/61)	75.0 (12/16)	70.0 (7/10)	100.0 (37/37)	ns	91.1 (113/124)
Resolution of megaesophagus (%, *n*)	53.3 (8/15)	28.6 (2/7)	50.0 (1/2)	50.0 (2/4)	ns	46.4 (13/28)
Treatment with AChEI (%, *n*)	81.9 (68/83)	65.6 (21/32)	80.0 (12/15)	100.0 (37/37)	N/A	82.6 (138/167)
Treatment with immunosuppressive drugs (%, *n*)	20.5 (17/83)	12.5 (4/32)	13.3 (2/15)	27.0 (10/37)	ns	19.8 (33/167)

*Note:* Superscripts indicate significant differences.

Abbreviations: AChEI, acetylcholinesterase inhibitor; ns, not significant.

The thymoma subgroup (median 102 months; Interquartile Range, IQR 96–120) was significantly older, and the seronegative subgroup was significantly younger (median 30 months, IQR 11.5–66) than the generalized subgroup (median 67 months, IQR 36–96 months) at the time of presentation (*p* < 0.001 and *p* < 0.017, respectively; Figure 1B). The median age of the focal group (median 68 months, IQR 27.8–108.8) was similar to that of the generalized group. In contrast to humans, there was no difference in gender between the young and old age.

The median duration of clinical signs before presentation varied from 4 to 17 days (IQR 2.3–27.8), depending on the subgroup, with no significant differences between the subgroups (Figure 1C).

### 3.4 Clinical Signs at First Presentation

As per the definitions, all dogs in the generalized subgroup exhibited generalized fatigable muscle weakness, compared to none of the dogs in the focal subgroup (Table 1). Most dogs in the thymoma (93.3%) and the seronegative (91.9%) subgroups presented with generalized weakness. One seropositive dog initially exhibited focal signs, but within a month, the signs progressed to generalized weakness (leading to its inclusion in the generalized group). Similarly, one seronegative dog initially showed focal signs, but within 2 weeks, the signs progressed to generalized weakness.

Of the 167 myasthenia cases, facial weakness was observed in 41.9% of dogs, laryngeal weakness in 22.2%, pharyngeal weakness in 42.5%, and esophageal weakness in 82.1% (Table 1). Muscle atrophy was observed in only 3.5% of all dogs. Pharyngeal weakness was significantly less prevalent in the seronegative group compared to the generalized group (21.6% vs. 53.0%; Odds' ratio 4.1, 95% confidence intervals (C.I). 1.6–9.7; *p* = 0.004; Table 1). Muscle atrophy was significantly more prevalent in the thymoma group compared to the generalized group (5.4% vs. 1.2%; Odds' ratio 12.6, 95% C.I. 1.3–184.1; *p* = 0.036).

### 3.5 Severity of Clinical Signs

**FIGURE 2:**
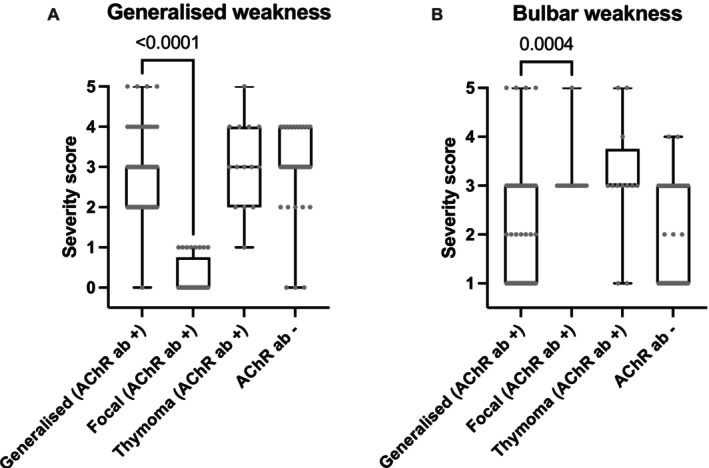
Severity scores of generalized (A) and bulbar weakness (B) were analyzed for the 4 myasthenic subgroups based on human MGFA scores (for interpretation of the scores, see methods).

The median severity grade of generalized weakness for the general, thymoma, and seronegative subgroups was 3 (IQRs 2–3, 2–4, and 3–4, respectively), indicating that rest was necessary for ambulation (Figure 2A). One dog from the generalized subgroup initially exhibited only focal signs at initial presentation, but later progressed to generalized signs. Grade 5 of generalized weakness (the ‘fulminant form’) was observed in four dogs of the generalized subgroup and one dog within the thymoma subgroup. The median bulbar severity score for all groups was 3 (IQRs 1–3, 3–3, 3–3.75, for generalized, focal, and thymoma subgroup, respectively), indicating frequent regurgitation, except for the seronegative group that had a median score of 1 (IQR 1–3), indicating no regurgitation (Figure 2B).

### 3.6 Diagnostic Tests

**FIGURE 3:**
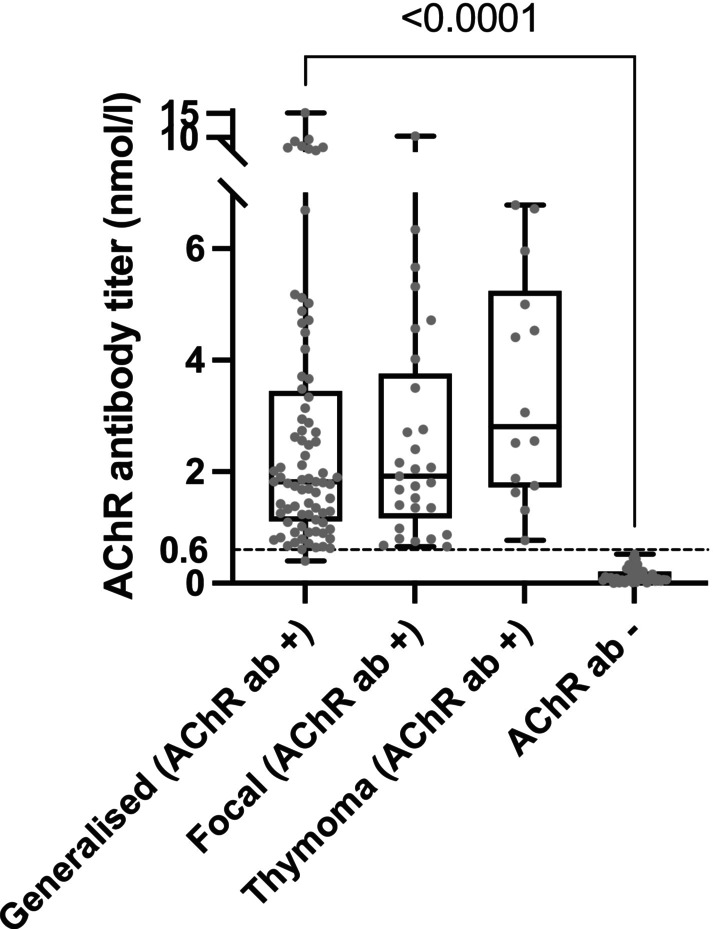
AChR antibody titers were analyzed for the 4 myasthenic subgroups.

In all 167 dogs suspected of having myasthenia gravis, the initial AChR RIA test was positive in 128 dogs (76.6%) and negative in 39 dogs (23%). In 10 seronegative dogs, the antibody test was repeated, with a median of 31 days between tests (range, 15–180 days). Two of these 10 dogs tested positive at 31 and 66 days, respectively. Therefore, these two dogs were categorized within the seropositive groups. The median AChR antibody titre for the generalized subgroup was 1.82 nmol/L (IQR, 1.11–3.45); for the focal group, it was 1.92 nmol/L (IQR, 1.17–3.76), and for the thymoma group, it was 2.81 nmol/L (IQR, 1.72–5.24). In contrast, the seronegative group had a median titre of 0.075 nmol/L (IQR, 0.05–0.21; Figure 3).

Most dogs underwent thoracic imaging, which typically involved radiographs and, in a few cases, computed tomography (CT), fluoroscopy, or a combination of these tests. Megaesophagus (or esophageal hypomotility) was documented in 84.1% of dogs, while changes consistent with aspiration pneumonia were observed in 55.4% of dogs (Table 1). Megaesophagus was significantly less prevalent in the seronegative group compared to the generalized group (63.8% vs. 85.7%; Odd's ratio 3.4, 95% C.I. 1.4–8.9; *p* = 0.025; Table 1).

Of 47 dogs that underwent electrodiagnostic testing, a decrement of more than 10% was observed during repetitive nerve stimulation in 46.8% (Table 1). Response to initial acetylcholinesterase inhibitors (typically edrophonium chloride or neostigmine methylsulphate response test, or initial empiric treatment with pyridostigmine in a few cases) was positive in 91.1% of all tested dogs (Table 1). Following the initiation of pyridostigmine, 28 dogs underwent repeated radiographs, and 46.4% of them showed a disappearance of megaesophagus.

### 3.7 Treatment

Most of the dogs in this study (82.6%) were treated with acetylcholinesterase inhibitors, typically per oral pyridostigmine, with a few dogs receiving parenteral neostigmine (Table 1). Most dogs that were not given acetylcholinesterase inhibitors died soon after presentation or before receiving a definitive diagnosis through the AChR RIA test, with a median duration of 3 days (range, 0–12 days). Two other dogs exhibited clinical signs that resolved spontaneously before the positive AChR RIA test results were obtained. One dog belonged to the generalized subgroup and had a serum AChR level of 6.69 nmol/L, while the other dog belonged to the focal subgroup and had a serum AChR level of 0.80 nmol/L. One dog (from the generalized subgroup) was subsequently diagnosed with insulinoma and its positive AChR antibody (0.64 nmol/L) was disregarded, so the dog did not receive pyridostigmine.

A fifth of dogs (19.8%) received immunosuppressants, typically prednisolone, with few cases involving other immunosuppressant drugs, such as azathioprine, cyclosporine, or mycophenolate (Table 1). All except one received this in addition to pyridostigmine. Four dogs with thymoma underwent thymectomy.

### 3.8 Comorbidities

All dogs belonging to the thymoma group had thymoma, and one dog within the seronegative group also had thymoma. We also recorded whether other comorbidities (immune‐mediated disease or neoplasia) than thymoma's were observed in any of the dogs. Hypothyroidism was diagnosed in two dogs, and masticatory myositis, trigeminal neuritis, steroid responsive meningitis, pemphigus foliaceus, and inflammatory bowel disease were diagnosed in each one dog. Malignant neoplasia was found in two dogs (splenic neoplasia, anal gland adenocarcinoma).

### 3.9 Survival and Progression Over Time

**FIGURE 4:**
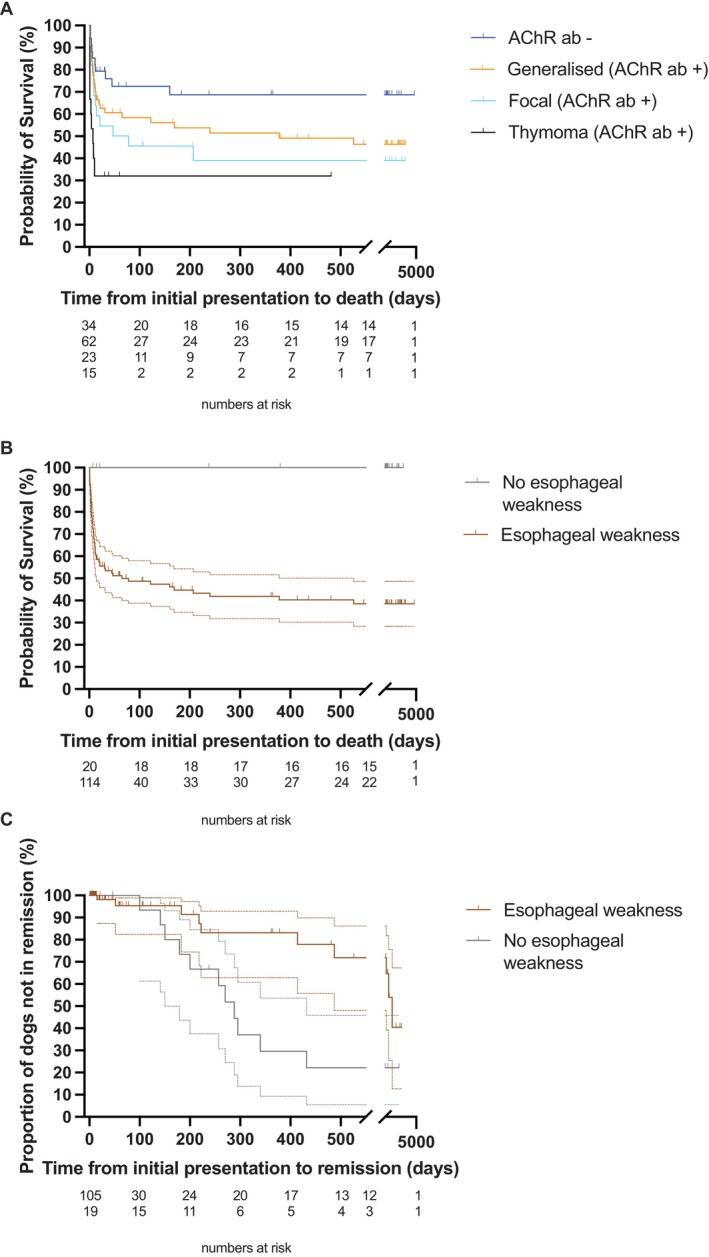
Survival curves and their 95% confidence intervals. (A) Survival time of the 4 myasthenia subgroups. Confidence intervals were omitted for clarity of the graph. (B) Dogs with esophageal weakness had shorter lifespans compared to dogs without esophageal weakness (*p* < 0.001). (C) Remission time. Dogs without esophageal weakness had a higher chance of remission from myasthenia (*p* = 0.005).

Outcome data was available for 134 cases. The median follow‐up period was 32 days, while the average was 551 days (ranging from 1, indicating death or euthanasia at presentation, to 4752 days). Survival times, calculated as the time elapsed between diagnosis and death or the last known time the dog was alive, were examined for the four myasthenia subgroups. The generalized subgroup had a median survival time of 378 days, the focal subgroup had a median survival time of 78 days, and the thymoma subgroup had a median survival time of 7 days (Figure 4A). The median survival time of the seronegative group could not be determined because many animals were still alive. The thymoma subgroup had a significantly lower survival time compared to the generalized subgroup (Hazard Ratio 3.69, 95% C.I. 1.38–9.88; *p* = 0.028).

The presence of esophageal weakness markedly reduced survival (Hazard Ratio 3.75, 95% C.I. 2.01–6.99; *p* < 0.001; Figure 4B). The median survival time when esophageal weakness was present was 65 days. In its absence, the median survival time could not be calculated, as all dogs were still alive.

The primary cause of death or euthanasia was aspiration pneumonia (52.1%, *n* = 38/73). Unrelated death was observed in 27.4% of cases (*n* = 20/73). Other reasons given were no effect of medication (4.1%, *n* = 3/73), persistence of regurgitation (2.7%, *n* = 2/73) or recurrence of signs (2.7%, *n* = 2/73), myasthenic or cholinergic crisis (2.7%, *n* = 2/73), respiratory distress (4.1%, *n* = 3/73), complications like sepsis following aspiration pneumonia (1.4%, *n* = 1/73) or peritonitis caused by a stomach tube (1.4%, *n* = 1/73), and thymoma (1.4%, *n* = 1/73). Costs were also often cited as a second reason for euthanasia.

During the follow‐up period, 18.2% of dogs diagnosed with myasthenia gravis experienced complete and sustained remission. None of the dogs were reported to relapse after remission. The median time to achieve remission was 712 days, which is equivalent to approximately 2 years. Remission was associated with a seroreversion to a negative AChR RIA titre, which was repeated during remission in seven cases. The sole predictor of remission time was the absence of esophageal weakness at the time of presentation (Hazard Ratio 3.794, 95% C.I. 1.43–10.07; *p* = 0.008; Figure 4C).

**FIGURE 5:**
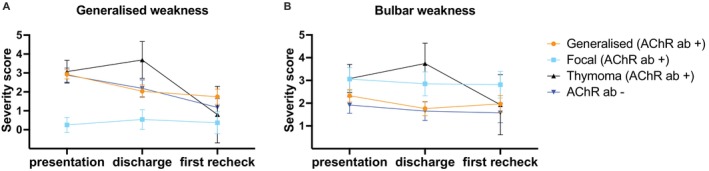
Severity scores of generalized (A) and bulbar weakness (B) were analyzed over time based on human MGFA scores (marginal means and 95% confidence intervals, linear mixed model). Three time points were examined: The initial presentation, discharge, and the first recheck. Generalized weakness significantly improved between the initial presentation and the first recheck (*p* = 0.012).

We analyzed severity scores over time for the four myasthenia gravis subgroups at initial presentation, at discharge, and at first recheck (Figure 5). Repeated severity scores could be determined for 108 dogs. The median discharge time was 4 days following presentation (range, 0–24 days), and the median first recheck time was 20 days (range, 3–88 days). Generalized weakness significantly improved between the initial presentation and the first recheck (*p* = 0.012). The mean scores for generalized, thymoma, and generalized subgroup at the initial presentation were 3, indicating that rest was required (95% C.I.s 2.5–3.7). In contrast, the mean scores for the first recheck were 0.8 to 1.7, depending on the subgroup, suggesting the absence of rest requirements for ambulation (95% C.I.s 0.7–2.3). Bulbar weakness did not demonstrate any statistically significant differences between the initial presentation and the first recheck in any of the subgroups.

## 4 Discussion

This large, multicentre study categorizes canine myasthenia gravis into distinct subgroups, including seronegative myasthenia gravis. Our research shows that (AChR antibody‐positive) generalized myasthenia has the highest prevalence, followed by seronegative, (AChR antibody‐positive) focal, and (AChR antibody‐positive) thymoma‐associated myasthenia gravis, in descending order. Dogs with thymoma were older, while seronegative dogs were younger. Seronegative dogs had a lower prevalence of megaesophagus. Esophageal weakness predicted survival and remission time.

The proportion of seronegative myasthenia gravis in our dog cohort (22.2%) is higher than previously reported (2%) [9]. The reported 2% is based on 1154 dogs testing positive for AChR antibodies and 20 dogs testing negative, but with typical clinical signs and a positive edrophonium test, during a defined time period [9]. Our higher (22.2%) proportion might be due to the availability of all clinical records and inclusion of electrophysiological studies and responses to all acetylcholinesterase inhibitors in seronegative cases (as per the human definition of seronegative myasthenia gravis [15, 20]). In humans, 10% to 20% of generalized myasthenia gravis cases and up to 50% of focal, ocular myasthenia gravis cases are also seronegative on AChR antibody RIA testing [12, 13, 14]. False positives are uncommon, as evidenced by our study, which identified one case that was considered a false positive.

Several factors, such as early sampling, the use of immunosuppressive drugs, and strong antibody binding to neuromuscular junctions, may explain seronegative AChR RIA results. However, the test's sensitivity and the presence of autoantibodies against non‐tested antigens are more likely causes [2]. For instance, in humans, it is well known that the RIA test may fail to detect low‐affinity AChR antibodies, while cell‐based assays can successfully detect them [12, 15]. Similarly, the AChR RIA test likely does not detect all cases of myasthenia gravis in dogs. Two studies support this: AChR‐antibody complexes were detected in muscle biopsies of 25% (*n* = 5/20) of dogs with typical myasthenia gravis symptoms and 11% of dogs (*n* = 17/152) with isolated esophageal weakness, despite negative AChR RIA test results [9, 19].

We strictly followed human definitions for seronegative myasthenia gravis [15, 20], aligning with the initial veterinary definition [11]. In veterinary medicine, a new definition was later proposed that included two subsequent negative RIA tests [9]. While anecdotal recommendations suggest repeating testing 1–2 months after a negative result in dogs [11], there are no veterinary publications studying repeated testing, such as the probability of a second test becoming positive and its timing. In our study, 10 dogs initially tested negative and were retested. Only two retested positive. This is similar to the human prevalence of 15%–18% of cases developing a positive AChR RIA test result later in the course of the disease, with some cases even becoming positive after nine years [25, 26, 27]. Repeated testing is not commonly conducted in humans, but a more sensitive test, like a cell‐based assay, is usually chosen. While we recommend a second RIA test for all seronegative dogs, financial constraints, the dog's positive response to treatment, or the dog's death can prohibit repeated testing, as exemplified in our study. If all dogs suspected of myasthenia gravis that initially tested negative for AChR antibodies had been tested twice with RIA (instead of just 10 dogs) and assuming 20% of them would have become positive, the seronegative group would still comprise 18.9% of the dogs. Further research is needed to explore the effectiveness of repeated testing with a larger sample size and repeated sampling over a longer period.

The AChR titre of the focal group was not lower than that of the generalized group, unlike a previous report [19]. Perhaps this is because we analyzed the results separately from thymoma‐associated myasthenia (in contrast to the previous report), a group that tended to have higher antibody titres in our study.

Seronegative dogs were younger and were less severely affected, similar to humans [16, 28, 29, 30]. The cause of the former is unknown [28].

The survival time varied among the four subgroups, with thymoma dogs surviving the shortest. Esophageal weakness significantly decreased survival and remission rates. In its presence, the median survival time was only 65 days, whereas dogs without esophageal weakness had a normal life span. The myasthenia gravis‐related mortality rate in dogs may be even higher than reported in this study, as 28 dogs suspected of having myasthenia gravis were excluded from this study because their AChR RIA test was either not performed or canceled due to their death or euthanasia shortly after presentation. Myasthenia gravis‐related mortality rates in dogs are undoubtedly higher than those observed in humans. This might be because myasthenic humans rarely present with megaesophagus. In addition, advancements in therapies, including steroids, immunosuppressive drugs, plasmapheresis, IV immunoglobulins, monoclonal antibodies targeting B‐cells, and complement inhibitors [2], have reduced myasthenia gravis‐related mortality rates to 3%–4% in human myasthenic patients [31]. These treatments are controversial in dogs with esophageal weakness due to the risk of aspiration pneumonia [10].

The remission rate in this study (18.2%) is comparable to a recent study (31%) [32], yet it differs from higher rates (88.7%) reported in a 2001 study [32]. The disparity might be attributed to the longer follow‐up period of 18 months or more in the 2001 study. By 18 months, more than 46% of the seropositive dogs in our study cohort would have already died (Figure 4).

Seronegative myasthenia gravis confirmation is controversial because specific diagnostic tests are lacking [33]. Our criteria followed human guidelines and included fatigable muscle weakness and a positive clinical response to acetylcholinesterase inhibitors or electrophysiologic decrement in the absence of a positive serum acetylcholine receptor antibody titre [15, 20]. In humans, the specificity of a beneficial response to acetylcholinesterase inhibitors is high (97%) for diagnosing myasthenia gravis [34]. Equally, the presence of a decrement with a cut‐off value of 10% has low sensitivity (29.5%), but high (96%) specificity [34, 35]. Although single‐fiber electromyography, a test rarely used in dogs due to technical challenges, is more sensitive than repetitive nerve stimulation [36], it has lower specificity (70%) [37]. Thus, while not all dogs with myasthenia may have decrement, as shown in our study (46.8%), it is unlikely we misdiagnosed them with myasthenia gravis in the presence of a decrement or beneficial response to acetylcholinesterase inhibitors.

Even with strict adherence to the above guidelines for classifying seronegative dogs with myasthenia gravis, it is possible that another neuromuscular disorder could have mimicked myasthenia in rare cases [33]. Therefore, further research and the development of novel serum antibody tests specifically for seronegative dogs are needed for a definitive diagnosis. Several antibody tests against multiple neuromuscular junction antigens have decreased the percentage of seronegative human patients. However, at least 5% of myasthenic human patients remain seronegative despite current testing [4].

On the other hand, in the absence of other autoantibody tests, we may have inadvertently excluded myasthenic dogs with a focal presentation since we only included dogs with megaesophagus exhibiting a positive decrement test, a positive AChR test, or a clear response to acetylcholinesterase inhibitors.

In conclusion, this study, by categorizing myasthenia gravis into subgroups, offers valuable insights for clinical decision‐making and prognosis. The age of onset, the presence of megaesophagus, and the survival times vary considerably among these subgroups. Moreover, by adhering strictly to human criteria, this study is a comprehensive description of seronegative myasthenia gravis cases in veterinary medicine.

## Disclosure

Authors declare no off‐label use of antimicrobials.

## Ethics Statement

Institutional Animal Care and Use Committee approval was granted for this study by the Royal Veterinary College (2023–2188) and Cambridge University (CR658). Authors declare human ethics approval was not needed.

## Conflicts of Interest

The authors declare no conflicts of interest.
